# The effectiveness of an online video-based group schema therapy in improvement of the cognitive emotion regulation strategies in women who have undergone bariatric surgery

**DOI:** 10.1186/s12893-023-02010-w

**Published:** 2023-04-27

**Authors:** Zahra Sobhani, Seyed Vahid Hosseini, Nazanin Honarparvaran, Hajar Khazraei, Masood Amini, Arvin Hedayati

**Affiliations:** 1 Colorectal research center, Shiraz University of Medical Sciences, Shiraz, Iran; 2 Department of counselling, Marvdash Branch, Islamic Azad University, Marvdasht, Iran; 3 Laparoscopy research center, Shiraz University of Medical Sciences, Shiraz, Iran; 4 Research Center for Psychiatry and behavior Science , Shiraz University of Medical Sciences, Shiraz, Iran

**Keywords:** Bariatric surgery, Group schema therapy, Cognitive emotion regulation, Body mass index

## Abstract

**Background:**

Adaptive cognitive emotion regulation (CER) strategies toward eating play a very important role in obesity and according to schema therapy, patients with obesity learn that don't respond to their emotional stimuli by eating. Thus, this study aimed to evaluate the effectiveness of an online video-based group schema therapy in improvement of the CER strategies and body mass index (BMI) in women who had undergone bariatric surgery.

**Methods:**

Forty women who had undergone sleeve gastrectomy were selected and randomly divided into two groups of control and experimental. The experimental group received 10 weekly 90-min sessions of group schema therapy, the control group did not receive any intervention at all. Both groups completed the CER strategies questionnaire during pre-test, post-test and follow-up stages, and the data were analyzed using a multivariate analysis of covariance (MANCOVA) through SPSS software (version 20).

**Results:**

Our results indicated that the experimental group demonstrated significantly higher adaptive CER strategies (*P* = 0.0001, F = 31.15) and significantly lower maladaptive CER strategies (*P *= 0.001, F = 9.42), significantly lower BMI (*P* = 0.001, F = 23.48), as compared to the control condition, following the group schema therapy after the follow-up stage.

**Conclusion:**

The findings demonstrated that group schema therapy could lead to an increases in adaptive CER strategies and a decrease in maladaptive CER strategies and BMI in women who had undergone bariatric surgery.

**Trial registration:**

IRCT, IRCT20180523039802N2. Registered 5 August 2020, http://www.irct.com/IRCT20180523039802N2.

## Introduction

Obesity is an epidemic disease and a major problem in public health; it affects over 1.5 billion people globally and is directly associated with co-morbidities such as arterial hypertension, diabetes, dyslipidemia, obstructive sleep apnea syndrome, fatty liver disease, and depression [1, 2]. Bariatric surgery (BS) is one of the most effective methods for the treatment of severe obesity that might lead to a long-term reduction in weight and can enhance the individuals’ psychological well-being and body image satisfaction [3, 4]. After BS, people are expected to abandon unhealthy eating behaviors such as emotional eating, eating at night, drinking high-calorie beverages, and returning to the pre-surgery lifestyle [5]. Many studies have documented the competence and effectiveness of bariatric surgery in reducing excess weight in the short-term, mid- and long-term with the maximum weight loss occurring at 12–18 months’ post-surgery, but some studies have reported weight regain as an index for failure of the surgery [6, 7]. A successful outcome of BS is achievement of a loss of 50–70% excess weight (EWL) or the 20–30% loss of the patient’s initial weight, or a BMI < 35 kg/m2 [6-8]; insufficient weight loss is defined as excess weight loss percentage (EWL%) of < 50% 18 months post-BS [9, 10].

Emotions directly influence thinking to facilitate decision-making, thinking, and action; accordingly, when experiencing negative emotions, they focus on more details and remember further errors. When experiencing positive excitement, they seek positive results and develop optimal performance [11]. Cognitive emotion regulation (CER) is defined as the “conscious, mental strategies individuals use to cope with the intake of emotionally arousing information” [12], and it involves four maladaptive and five adaptive strategies. The four maladaptive CER strategies are rumination, self-blame, blaming of others and catastrophizing; they can lead to psychological and emotional problems such as depression, anxiety or risky behaviors like overeating [12-14]. By contrast, positive refocusing, refocusing on planning, acceptance, putting into perspective and positive reappraisal are the five adaptive strategies that are related to better mental health and well-being [13]. Emotions, attitudes, and behaviors toward eating play a very important role in obesity, and negative emotions can be a powerful predictor of overeating; also, emotional eating lead to poor postoperative weight loss [15-17]. Eating behaviors are connected to the balance of the different features of mental dimensions implicated in the emotional regulation system and could provide significant clinical information and therefore it should be a part of the obesity diagnostic criteria and therapeutic programs [13]. Women with overweight and obesity, in comparison to the healthy group, are less aware of their emotions and have difficulty regulating their emotions, and feelings of loneliness or embarrassment which leads to binge eating [18].

Schema Therapy (ST) is an integrated cognitive-behavioral and emotion-focused model that links the actual psychological features and problems to childhood experiences; it also considering the role of temperamental features, helps to understand and treat personality disorders [19, 20] and long lasting emotional and interpersonal problems. It might be useful to heal chronic behavioral problems, such as those related with obesity. The key concepts within the ST approach include early maladaptive schemas (EMSs), schema modes, and dysfunctional coping strategies [21]. The goal of psychological therapy for obesity is training the clients to differentiate between emotional hunger and physiological hunger that do not respond to their emotional stimuli by eating [15]. According to a schema-based approach, patients with obesity learn some reactions and coping strategies since childhood, such as avoiding experiencing intense emotions that resulted from early maladaptive schemas, and they are more likely to suffer from negative self-esteem [19, 22, 23]. Maladaptive schemas can cause overeating to reduce negative emotions [16] and dysfunctional beliefs about body shape, weight, and eating issues are important variables in obesity [20, 21]. Some of the early maladaptive schemas related to obesity are abandonment, emotional deprivation, mistrust/abuse, social isolation, dependence defectiveness/shame and insufficient-self-control. They are considered to have developed through the interaction between child temperament and early experiences of deprivation and/or frustration [21, 24].

Mental health and eating behaviors may improve after BS, and these beneficial effects may have an effect on weight reduction and maintenance [25]. Nevertheless, these advantages do not appear to endure beyond the years after surgery [26]. While studying the need for implementing CER strategies in women who underwent BS seems necessary in the modern world of today, research on the subject and its.

Application is still in its infancy as well. Some studies demonstrated that cooperation in group-based psychotherapy positively affected weight loss results among post- BS patients [27, 28]. Therefore, psychotherapy may be helpful to improve CER strategies in patients with obesity. After BS, some individuals failed in attaining or keeping up ideal weight reduction, and it is far said that 18% of 500 bariatric applicants failed to attain excess weight loss after bariatric surgery that one of the foremost commonplace reasons is overeating to manage emotional distress [1, 6]. Emotional eating or eating in reaction to emotional distress is related to suboptimal weight reduction and has been found to comply with bariatric operations [29]. So far, the effects of various psychotherapies such as cognitive-behavioral therapy (CBT) [30-33], motivational interviewing (MI) [34, 35], Information-Motivation-Behavioral model (IMB-model) [36], and acceptance and commitment therapy (ACT) [37] have been conducted for post-BS patients, but we could not find any research that has examined the effect of schema therapy on improvement of the CER strategies in these patients. Some studies show that schema therapy compared to other psychotherapies such as CBT had high effectiveness on eating disorders [38]. It may be because schema therapy which is an emotion-focused model and a combination from CBT, psychoanalysis, attachment and object relations theories, which is more effective for the treatment of obesity; also, group schema therapy probably improves the eating disorder symptoms [20, 39, 40]. Therefore, this study aimed to evaluate the effectiveness of an online video-based group schema therapy on improvement of the CER strategies in women who had undergone BS.

## Material and methods

### Participants

This randomized clinical trial was registered in the Iranian Clinical Trial registry under number IRCT 20180523039802N2. The target population consisted of all women whose BMI was higher than 35 (mean ± SD = 38/47 ± 23/54) and aged 25 to 45 years old (mean ± SD = 36.14 ± 10.32) who underwent sleeve gastrectomy in 2020 during the COVID-19 period. The inclusion criteria were receiving sleeve gastrectomy between 18 to 24 months ago, having insufficient weight loss and BMI between 35 to 40 after surgery, and having comorbidities and any psychological disorders such as depression [4, 10, 30].

### Study design and randomization

**Fig. 1:**
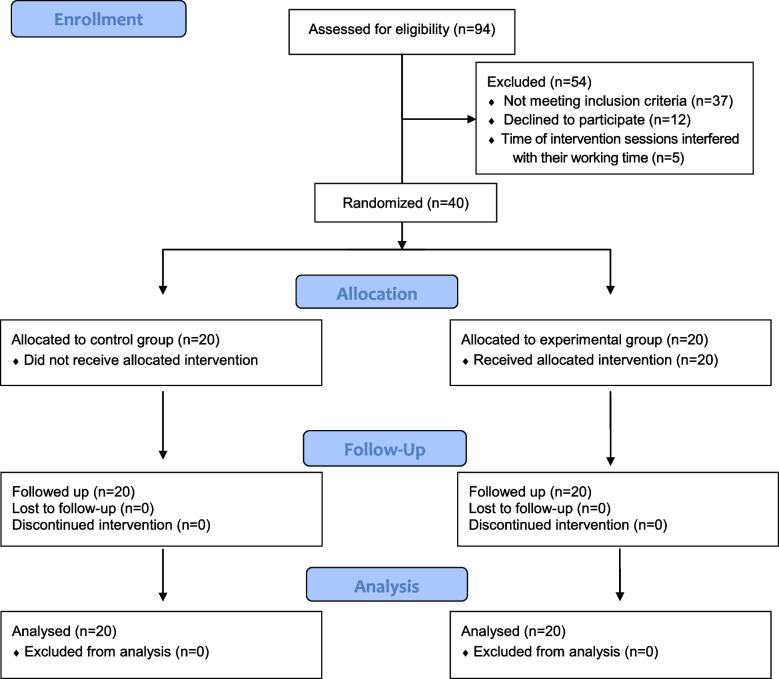
CONSORT 2010 flow diagram is depicted

We assessed 94 patients at first but 54 patients were excluded from study; 37 patients did not meet the inclusion criteria; 12 of them withdrew from the study and 5 patients were excluded because the time of therapy sessions interfered with their working time (Consort flow diagram). Based on the formula of the sample size comparison of two means with an error of 5%, with a power of 90%, and using the results of a similar study [30], the sample size in each group was calculated 20 patients and no drop out during the treatment period. Accordingly, 40 patients who entered the study were randomly divided into two groups (experimental and control), each with 20 with block randomization. The randomization list was generated and used using random allocation software. There was no blinding in this study (Fig. 1).

The research objectives were explained to all the participants. Written and signed consent form was obtained from the participants. Additionally, all the participants in the study were assured that their information would remain confidential and that researchers would only report the group results of the study. The experimental group received 10 weekly sessions of group schema therapy with 90 min per session, but the control group did not receive any intervention. Schema therapy was performed by the research team psychologist who was expert in health psychology and had a certificate of specialized schema therapy courses. Both groups completed CER questionnaire during pre-test, post-test, and follow-up stages (follow-up time point was 6 months after post-test). The data were analyzed using a multivariate analysis of covariance (MANCOVA) in SPSS software (version 20).

### Interventions

The study intervention consisted of 10- weekly sessions (90 min) of group schema therapy which were held online (because of COVID-19 pandemic) with the zoom meeting program. It was based on the Young et al.’s schema therapy protocol [19]. Briefly, this psychological treatment focused on understanding the developmental origins of the schema and relating it with obesity and relating them with currently used cognitive emotion regulation strategies, and then using the advantage-disadvantage technique to decide on whether or not to keep a schema and teaching emotion regulation techniques [27, 39, 40] (Table 1).

**Table1:** Content of the group schema therapy sessions

Sessions	Content of the Sessions
**1**	Introducing and framing the problems in schema therapy format
**2**	Selecting the emotional inhibition and the emotional deprivation schemas and understanding fundamental beliefs
**3**	Exploring the developmental origins of the schemas in childhood and relating them with currently-used coping strategies
**4**	Assessing reasons behind the formation of schemas, particularly in the interactions with parents, based on the assumptions of schema therapy
**5**	Schema testing and analyzing them through Socratic dialogue; reframing the schemas
**6**	Implementing limited reparenting to recognize participants’ needs
**7**	Exploring the origins of the schemas through experiential techniques;
**8**	Understanding the developmental origins of the schema and relating it with obesity
**9**	Using the advantage-disadvantage technique to decide on whether or not to keep a schema and teaching emotion regulation techniques
**10**	Assessing treatment outcomes and the methods for maintaining them through flashcards

### Measurements

#### Cognitive emotion regulation strategies questionnaire (CERQ)

The Cognitive Emotion Regulation Questionnaire (CERQ) is a 36-item self-report measure designed to assess individual differences in cognitive regulation of emotions in response to stressful, threatening, or traumatic life events. It contains nine conceptually distinct subscales: five adaptive strategy subscales (acceptance, positive refocusing, refocusing on planning, positive reappraisal, and putting into perspective) and four maladaptive strategies subscales (self-blame, rumination, catastrophizing, and blaming others). Responses to the items are measured on a five-point Likert scale with a range from 1 [(almost) never] to 5 [(almost) always]; a higher subscale score indicates greater use of a specific cognitive strategy. The CERQ has shown excellent reliability and validity [12, 41]. The Cronbach’s alpha reliability coefficient of adaptive and maladaptive and total scores of CERQ was found to be 0.91, 0.87, and 0.93, respectively. All subscales have good internal consistencies ranging from 0.68 to 0.86 [42]. In Iran, the results of Cronbach's alpha were in the range of 0.68 to 0.82, indicating that the 9 subscales of the questionnaire have good reliability [43]. The range of Cronbach's alpha in the present study was 0.74 to 0.79, which shows an acceptable reliability of the questionnaire.

#### BMI measurement

Body mass index (BMI) is a measure of weight adjusted for height, calculated as weight in kilograms divided by the square of height in meters (kg/m2) [44]. Therefore, in this research BMI was calculated based on this formula:

$$\mathrm{BMI}\left(\mathrm{kg}/\mathrm{m}2\right) =\frac{\mathrm{Weight}\left(\mathrm{kg}\right)}{\mathrm{Height}\left(\mathrm{m}\right)2}$$

### Data analysis

The data were analyzed using SPSS version 20 in the present study. To examine the main hypothesis in the current study, we used analyses of covariance's (MANCOVAs) by group status (as fixed independent variable), BMI and CER strategies as dependent variables. Furthermore, baseline measures of BMI, adaptive CER strategies and maladaptive CER strategies were considered as covariates. The significance level for hypothesis testing was *P* < 0.05.

### Ethics

The study was approved by the Ehtics Committee of Shiraz University of Medical Sciences (no.IR.SUMS.REC.1398.237), and it satisfies the Declaration of Helsinki standard [45].

## Results

Table 2 shows the mean and standard deviation of BMI, adaptive CER strategies, and maladaptive CER strategies scores in the experimental and control groups in pre-test, post-test and 6-month follow-up stages (Table 2). The descriptive data obtained in Table 2 indicate that the mean and standard deviation of BMI, adaptive CER strategies, and maladaptive CER strategies scores in the experimental and control groups in pre-test, post-test and follow-up stages are not the same. The scores of the experimental group in the post-test and follow-up stages improved after the schema therapy sessions.

**Table 2:** Mean and standard deviation of dependent variables in pre-test, post-test and follow-up stages among experimental and control groups

Variables	Stages	Experimental Group		Control Group	
		**Mean**	**SD**	**Mean**	**SD**
**BMI**	**Pre-test**	38.23	6.75	38.45	7.13
	**Post-test**	36.48	6.24	37.82	6.38
	**Follow-up**	34.12	5.87	38.23	6.74
**adaptive CER strategies**	**Pre-test**	52.31	7.89	51.68	8.37
	**Post-test**	65.42	8.32	52.24	9.46
	**Follow-up**	64.87	7.69	51.98	8.58
**maladaptive CER strategies**	**Pre-test**	51.43	8.57	50.96	9.25
	**Post-test**	39.52	8.96	49.33	8.97
	**Follow-up**	40.65	9.23	50.76	8.45

*CER* Cognitive Emotion Regulation, *BMI* Body Mass Index

To run the main statistics, at first we computed all basic normality assumptions which showed a normal distribution for running of MANCOVAs in this study. Kolmogorov–Smirnov and Shapiro-Wilks tests of normality showed normal distribution for adaptive BMI, CER strategies, and maladaptive CER strategies as dependent variables during pre-test, post-test, and 6 months’ follow-up stages (Table 3).

**Table 3:** Test of normality for dependent variables during pre-test, post-test and follow-up stages

Stages	Variables	Kolmogorov–Smirnov test		Shapiro-Wilks test	
		**Statistic**	**p**	**Statistic**	**p**
**Pre-test**	**BMI**	0.68	0.112	0.896	0.127
	**adaptive CER strategies**	0.84	0.124	0.842	0.104
	**maladaptive CER strategies**	0.97	0.095	0.912	0.178
**Post-test**	**BMI**	0.034	0.192	0.894	0.766
	**adaptive CER strategies**	0.068	0.183	0.945	0.842
	**maladaptive CER strategies**	0.092	0.197	0.932	0.811
**Follow-up**	**BMI**	0.124	0.163	0.945	0.357
	**adaptive CER strategies**	0.082	0.177	0.914	0.345
	**maladaptive CER strategies**	0.114	0.159	0.961	0.468

*CER* Cognitive Emotion Regulation, *BMI* Body Mass Index

The effect of schema therapy on BMI and CER strategies are presented in MANCOVA. In the current study before performing MANCOVA, the presumptions were firstly tested such as the Box test and Levine's test. Box’s M test for the equality of covariance across experimental and control groups showed the normal distribution of BMI and CER strategies as the basic assumption of MANCOVA (F = 0.66 = 1.86, *p* = 0.72), and according to Levine's test and the significance level was (*P > 0.05).

Therefore, covariance analysis can be used to examine the data. Multivariate analysis of covariance was used to test the hypothesis that schema therapy influences the BMI and CER strategies after BS with 6 months’ follow-up. The results are presented in Tables 4 and 5.

**Table 4:** Effect of Eta based on Pillai’s trace and Wilk's lambda test for combinational variable

Variable	value	F	df Hypothesis	df error	Eta	Test Power	*P*-value
**Pillai’s Trace**	0.76	8.23	8	12	0.79	0.98	0.001
**Wilk’s Lambda**	0.34	8.23	8	12	0.79	0.98	0.001

**Table 5:** The level of BMI and adaptive cognitive emotion regulation (CER) strategies and maladaptive cognitive emotion regulation (CER) strategies in experimental and control groups

Variables	Sum of squares	DF	Mean of squares	F	*P*-value*	Eta	Test power
**BMI**	34.76	1	34.76	23.48	0.001	0.325	0.99
**adaptive CER strategies**	114.832	1	114.832	31.15	0.0001	0.574	0.97
**maladaptive CER strategies**	25.428	1	25.428	9.42	0.001	0.342	0.78

*Multivariate Analysis of Covariance (MANCOVA)

Table 4 shows that there is a significant difference between the experimental and the control groups in terms of the dependent variables (*p* < 0.001) and the observed power of 0.98. Accordingly, it can be stated that there is a significant difference between the two groups in at least one of the dependent variables (BMI, adaptive, and maladaptive cognitive emotion regulation strategies). To discover this difference, we used MANCOVA (Table 5). Moreover, the effect size coefficient indicated that probably the 0.79 difference between these two groups was the result of schema therapy intervention after 6 months’ follow-up.

According to Table 5, the differences between the experimental and control groups during pre-test, post-test, and 6 months’ follow-up stages in variables of BMI (*P* = 0.001, F = 23.48) adaptive CER strategies (*P* = 0.0001, F = 31.15), and maladaptive CER strategies (*P* = 0.001, F = 9.42) were significant. Our results indicated that the experimental group demonstrated significantly higher adaptive CER strategies and significantly lower maladaptive CER strategies and significantly lower BMI, compared to the control condition, following the intervention, which means that maybe schema therapy increases in adaptive CER strategies as well as a decreases in BMI and maladaptive CER strategies.

## Discussion

The aim of this study was to evaluate the effectiveness of an online video-based group schema therapy on the BMI and cognitive emotion regulation (CER) strategies in women who underwent bariatric surgery. Our results indicated that the experimental group demonstrated significantly higher adaptive CER strategies (*P* = 0.0001), significantly lower maladaptive CER strategies (*P* = 0.001), and significantly lower BMI (*P* = 0.001), compared to the control condition, following the group schema therapy during 6-month follow-up. Based on the current study findings, probably ST was effective in improving BMI and CER strategies in women who had undergone sleeve gastrectomy. The reason for the effectiveness of ST in CER strategies is the mechanisms in this treatment. In this process, complete identification of the patient’s emotions and their adjustment, identifying incompatible emotional strategies, and replacing them with more compatible emotional strategies, paying attention to the patient’s emotions and using them to deepen the treatment and the importance of therapeutic relationship are performed [46]. One possible explanation could be that schema therapy focused on emotions and childhood problems, and there is a relationship between psychological factors on weight status, BMI, especially cognition-emotional self-regulation, psychological well-being, and body image satisfaction [9, 10, 12, 20]. The effectiveness of schema therapy on improving CER strategies has not been studied so far, but probably group schema therapy was effective for improvement of eating disorders, and performing schema therapy for people with obesity shows that the severity of eating disorders may be related to early maladaptive schemas [22, 23, 40].

The major challenge to the successful outcome of bariatric surgery is in maintaining weight loss in a long-term and minimization of the weight regain [47]. Despite successful weight loss after BS, weight regain (WR) may occur on long term following most bariatric procedures, with 20–30% of patients either failing to reach their target weight goals or failing to maintain the achieved weight loss. Significant WR has important health consequences, reverses the improved obesity-related comorbidities and psychological function leading to decreased quality of life. Given the challenges faced by these patients, there is a need for multidisciplinary approaches to deal with WR [10, 48]. Early maladaptive schemas (EMSs) are core characteristic have been considered to have progressed early in childhood through the interaction between the child temperament and the early experiences of deprivation and/or frustration related to eating psychopathology, so that patients with obesity can avoid experiencing strong emotions ensued from early maladaptive schemas [36]. A negative perception of themselves leads to activating the abandonment schema, thus increasing food consumption as a means to avoid the negative emotions that result from it. These results are also similar to those of Peltzer et al., stating that behaviors such as overeating are designed to reduce negative emotions caused by maladaptive schemas [16]. Bamelis et al. showed that schema therapy was designed to help each person eliminate negative patterns of thinking, feeling, and behavior and make more beneficial choices to replace them [24]. The ability to manage and accept emotions and to act according to the intended goals is very important to to achieve weight loss after bariatric surgery [5, 10]; in this study, we made an attempt to improve the CER strategies in women who had undergone sleeve gastrectomy by group schema therapy. In the same vein, Chesler proposed that untreated emotional eating was a risk factor for poor postoperative weight loss [17].

Several studies have shown the effectiveness other psychotherapies such as CBT, MI, IMB model and ACT on weight regain, emotional eating, disordered eating behaviors, adherence, body size, depression, and obsessive–compulsive disorder in post-bariatric surgery patients [30-37]. For example, in Paul et al.’s study, CBT seems to be effective in reducing the risk factors for weight regain after BS, such as disordered eating behavior and depression [31]. The findings of Hosseini et al.’s study demonstrated that CBT could lead to reduce symptoms of obsessive–compulsive disorder (OCD) in patients who had undergone BS during COVID-19 [32]. In the same line, Rudolph and Hilbert reported the effectiveness of CBT for patients with behavior eating disorder after BS [33]. According to the results of Sobhani et al. MI can be implemented to enhance adherence and self-management behaviors, achieve favorable weight loss, and reduce postoperative complications in obese patients who had undergone sleeve gastrectomy surgery [34]. David et al. also suggested that MI was a satisfactory and practical intervention that could improve the patients' confidence to change their eating behaviors [35]. Khosravi et al.’s results showed that IMB model could be effective on improving weight and body size in women who have undergone bariatric surgery [36]. Cotter pointed out that acceptance and commitment therapy, as well as group therapy, was satisfactory for most of the patients who experienced the bariatric medical procedure, and could provide optimal effectiveness [37].

But given the limited efficacy of the maintenance of other models, there has been increased attention dedicated to exploring the role of deeper level factors such as core beliefs and schemas in the eating disorders literature. Schema therapy, is an emotion-focused model which is more effective for the treatment of childhood disorders like obesity because it is a combination of other theories [19]. In the same vein, Mahmoudian Dastnaii proposed that schema therapy compared to other psychotherapies such as CBT, was highly effectiveness on eating disorder [38]. Also Simpson et al. reported that group schema therapy highlighted the emotion regulation skills which had a significant alteration in early maladaptive schemas, a decrease in feelings of shame, and anxiety in females with obesity [40], so that schema therapy can be considered as an important line of treatment for adults with eating disorders and can guide psychotherapists to deliver ST for EDs confidently and successfully [39]. Thus, the results of the present study also support their findings, and probably schema therapy is effective in improving BMI and CER strategies in women who had undergone sleeve gastrectomy. According to the mentioned results, most medical interventions for reducing weight have so far ignored the role of psychological factors; it is recommended that group schema therapy, which can be convenient and probably effective on the CER strategies in patients who have undergone BS, should be implemented for these patients. However, definitive comments, in this regard, more studies are required longer follow-up and it appears that interventions are necessary to increase the rate of BMI.

Finally, it can be admitted that like any other studies, the current research had some limitations. For instance, due to the corona pandemic, the intervention was held online and we could not have individual schema therapy. Also, our statistical population was only in the city of Shiraz that may stand in the way of the generalizability of its findings. Another limitation was that due to cultural and social issues the participants in this study were only females, because losing weight for women has a lot of attention in Iran, but it is not considered a special advantage for Iranian men. Therefore, it is recommended that future studies should include both males and females to compare gender differences in the outcome. For more effectiveness of ST in post-corona period studies, interventions should be held face-to-face. Also, it is recommended that to compare findings from individual vs. group schema therapy in future studies, because a supportive group environment could help people improve their skills in a number of ways. Also it is suggested that ST should be performed for patients who have undergone BS in other cities and countries as well.

## Conclusion

The findings demonstrated that group schema therapy training could lead to improve BMI and CER strategies in women who have undergone BS. Maladaptive CER strategies can cause uncontrolled negative emotions that lead to overeating and increase in BMI. Thus, weight management programs for patients who have undergone BS should focus on improving their control of situations related to negative emotions by improving the CER strategies.

## Data Availability

All data generated or analyzed during this study are included in this published article.
